# Supplementary material for: Simple descriptor derived from symbolic regression accelerating the discovery of new perovskite catalysts
Source: Nat Commun. 2020 Jul 14;11:3513. doi: 10.1038/s41467-020-17263-9 (PMC7360597; doi:10.1038/s41467-020-17263-9)
Supplement: Supplementary file 1 — Supplementary Information [file 41467_2020_17263_MOESM1_ESM.pdf]

Supplementary Information

Simple Descriptor Derived from Symbolic Regression Accelerating the Discovery of New Perovskite Catalysts

Baicheng Weng#, Zhilong Song#, Rilong Zhu et al.

Simple Descriptor Derived from Symbolic Regression Accelerating the Discovery of New Perovskite Catalysts

Baicheng Weng[1,2,3,#], Zhilong Song[2,#], Rilong Zhu[4], Qingyu Yan[4], Qingde Sun[2], Corey G. Grice[1], Yanfa Yan[1,*], Wan-Jian Yin[2,5*]

[1]Department of Physics & Astronomy, and Wright Center for Photovoltaics Innovation and Commercialization, The University of Toledo, Toledo, OH 43606, USA

[2]College of Energy, Soochow Institute for Energy and Materials InnovationS (SIEMIS), and Jiangsu Provincial Key Laboratory for Advanced Carbon Materials and Wearable Energy Technologies, Soochow University, Suzhou 215006, China

[3]College of Chemistry and Chemical Engineering, Central South University, Changsha 410083, China

[4]College of Chemistry and Chemical Engineering, Hunan University, Changsha 410082, China

[5]Key Lab of Advanced Optical Manufacturing Technologies of Jiangsu Province & Key Lab of Modern Optical Technologies of Education Ministry of China, Soochow University, Suzhou 215006, China

Supplementary Note 1

Definition and abbreviation of variables

r_A weighted averaged ionic radii of cation A
r_B weighted averaged ionic radii of cation B
r_O ionic radii of anion O^{2-}
χ_A weighted averaged atomic radii of A
χ_B weighted averaged atomic radii of B
Q_A weighted averaged charged state of cation A
N_d weighted average number of *d* electrons at atom B
μ octahedral factor, r_B/r_O
t tolerance factor, $\frac{r_A+r_O}{\sqrt{2}(r_B+r_O)}$

Note: for oxide perovskites $(A^1_x A^2_{1-x})(B^1_y B^2_{1-y})O_3$, the weighted averaged ionic radii of cation A is $r_A = x \cdot r_{A^1} + (1-x) \cdot r_{A^2}$.

Supplementary Methods

Symbolic regression (SR) [1-4] simultaneously searches for the optimal mathematical formula of a function and set of parameters in the function. Therefore, it may overcome the issue of hidden black-box and[5] make machine learning (ML) model interpretable. In this work, symbolic regression is to build straightforward and effective descriptors that are able to link the easily-accessed materials parameters with catalytic activities. There are three essential parts for SR to derive descriptors: primitive function, terminal, and evolutionary algorithm.–

Primitive function

The primitive functions are the basic building blocks of mathematical formulas. It may contain mathematical operators (+, −, ×, ÷) and common functions ($\sqrt{}$, sin, cos, exp, log …). To ensure that the final derived descriptors are as simple as possible, we use only simple mathematical operators and functions in this work, including (+, −, ×, ÷, $\sqrt{}$).

Terminal

The terminals are variables and constants in mathematical formula. In this work, I should include key materials parameters relevant to the catalytic activity. In this work, we chose the terminal set (N_d, χ_A, χ_B, r_A, Q_A, t, μ) (Q_B and r_B are trivially dependent on Q_A and μ, respectively, subject to charge balance $Q_A + Q_B = 6$ and $r_B = \mu r_O$). For double perovskite and perovskite alloys, the weighted averages of N_d, χ_A, χ_B, Q_A, and r_A were taken for A- and B-site cations respectively, and t and μ were calculated based on the averaged r_A and r_B values. The values of terminal sets for all perovskites of interest in this work are provided in Tab. 1 and the details for calculating averaged values are provided in Supplementary Table 7.

Evolutionary algorithm

The available primitive functions and terminals provide the basis to compose simple and accurate mathematical formula correlating materials parameters (N_d, χ_A, χ_B, r_A, Q_A, t, μ) and catalytical activity (V_{RHE}). Evolutionary algorithm is the computational algorithm composing primitive functions and terminals into mathematical formula. It may include genetic programming, grammar evolution, and analytical programming and Bayesian optimization. Here, we chose genetic algorithm (GP) for SR (GPSR) as it is the mostly-used algorithm and implemented in *gplearn* code.

In GPSR, the mathematical formulas are expressed as tree structures which are composed of primitive functions and terminals (see examples in Supplementary Figure 12). The flowchart of SR can be found in Fig. 2b (simplified version) and Supplementary Figure 11 (detailed version). It initially builds a population (N_{ind} =5000) of random mathematical formulas composed of primitive functions and terminals, *i.e.*, a random tree structure with random nodes, to represent relationships between materials parameters and catalytic activity (V_{RHE}) [Fig. 2b]. The accuracy of 5,000 mathematical formulas are evaluated by mean absolute errors (MAE) between formula-fitting and experimental V_{RHE}s for eighteen known perovskites [box 2 in Fig. 2b]. The algorithm selects the best formula (least MAE) into the pool of final solutions set [Fig. 2b]. To generate the next generation, a fraction of mathematical formulas (here 1000 formulas) are selected by using a tournament method (with tournament size 20) as implemented in gplearn[6]. Genetic operations of crossover and mutation are then performed among them [box 5 in Fig. 2b] to form 1000 new ones for the next generations [Fig. 2b]. Examples of crossover and mutation operations are shown in Supplementary Figure 10. Another 4000 random formulas are then added to supplement new generation up to 5000 formulas in total [Fig. 2b]. The best formula in new generation is then selected [Fig. 2b] into the final solution set [Supplementary Figure 2b]. In principles, the procedure continues until a good formula with desired function metric (MAE < 0.01 eV) is found or the maximum generation reaches N_{maxG} = 20 [Fig. 2b].

Grid search of hyperparameters

In GPSR, the results may depend on how crossover and mutation are performed on 1000 selected formulas in each generation. To mitigate the impact of artificial hyperparameters on final

results, we used grid search of hyperparameters. The grid search method was used for hyper-parameters of *pc*, *ps* and *parsimony coefficient*, as shown in the Tab. 3. There are 18 *pc* values from 0.5 to 0.95 with step of 0.025, 8 *ps* values and 3 parsimony coefficients. Therefore, a grid search contains 18 × 8 × 3 = 432 combinations of hyper-parameters. In each combination of hyperparameters [box 0 in Fig. 2b], there are maximum 20 generations and each generation produces one best individual, which results in about 8,640 individuals. The Pareto front, showing the trade-off between the MAE and complexity, of total 43,200,000 individuals (8640 generations × 5000 individuals/generation) is shown in Fig. 3a with density plot.

Supplementary Figures

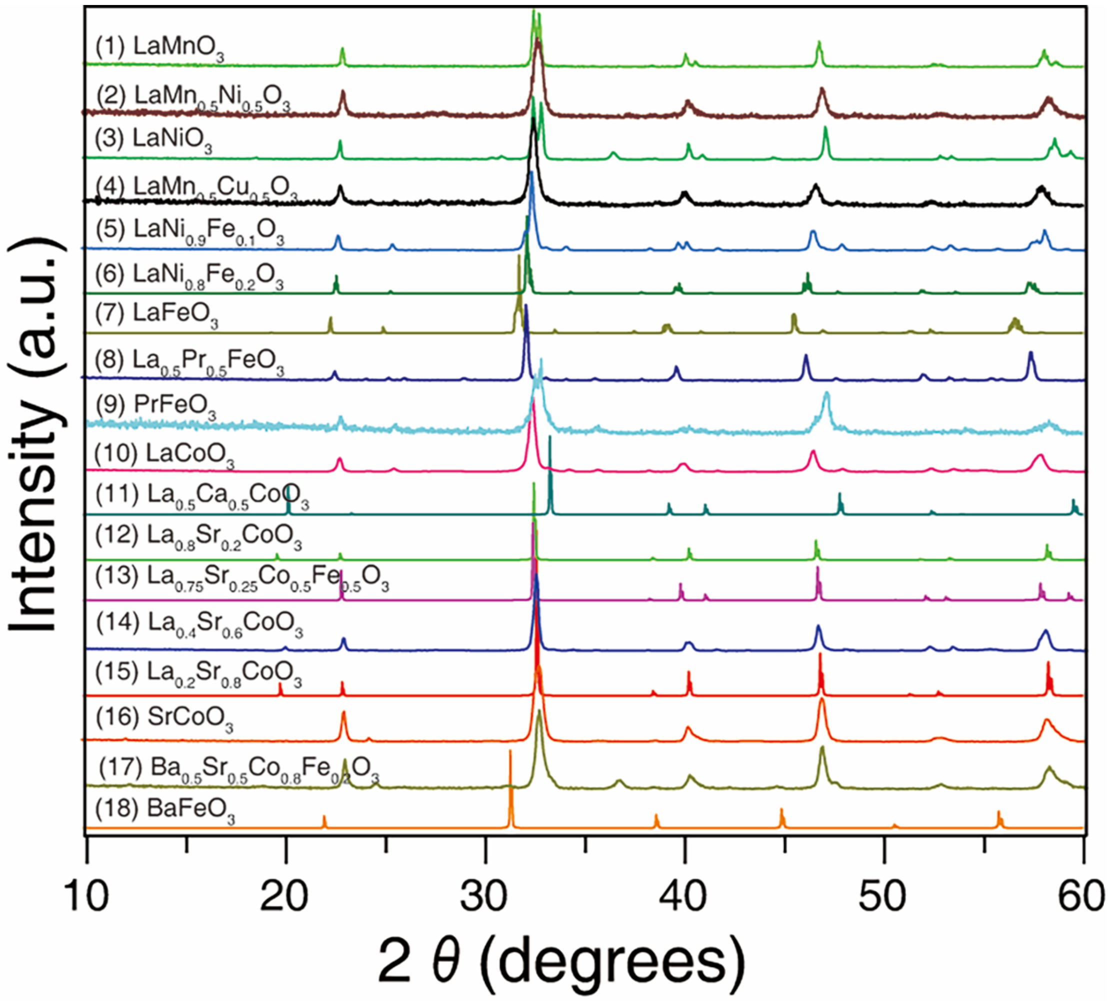

Supplementary Figure 1 PXRD data of 18 known oxide perovskites. The first characteristic peak of a perovskite structure around 20–25° corresponds to (100) facet, the main peak at 30–35° corresponds to (110) facet and 40° corresponds to (111) facet, 45–50° and 55–60° correspond to (200) and (211) facets, respectively. Here we cannot exclude the possible existence of oxygen vacancies that usually exist in oxide perovskite. For clarity, stoichiometric chemical formulas are adopted in this work.

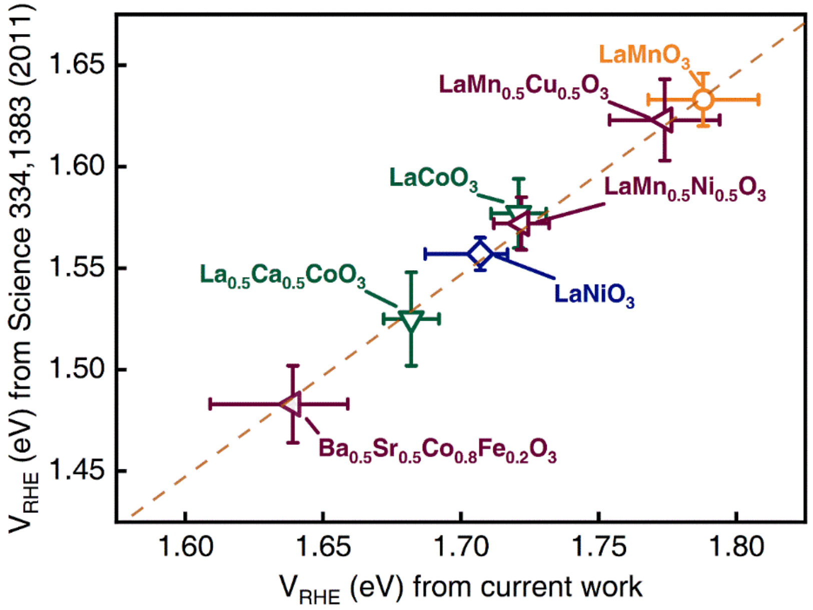

Supplementary Figure 2 Comparison of V_{RHE}s of seven oxide perovskite OER catalysts. The results from this work (at 5 mA/cm^2) are presented in the horizon axis and the results reported by Suntivich *et al.* [Science 334, 1383 (2011)] are presented in the vertical axis.

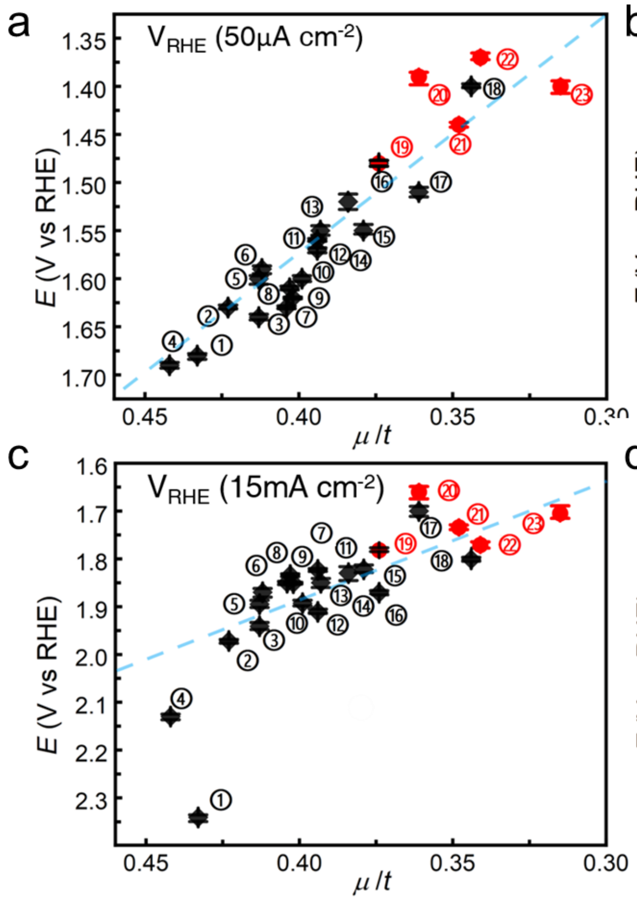

Supplementary Figure 3 The V_{RHE}s dependence on *μ/t* at current densities of **a,** 50 μA/cm^2. **b,** 10 mA/cm^2. **c,** 15 mA/cm^2. **d,** 20 mA/cm^2.

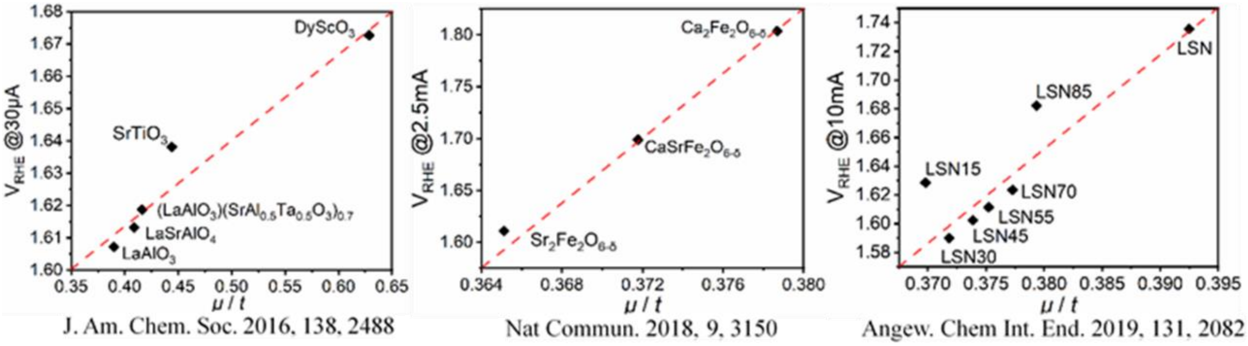

Supplementary Figure 4 V_{RHE} values, shown according to μ/t, sourced from three recent publications.

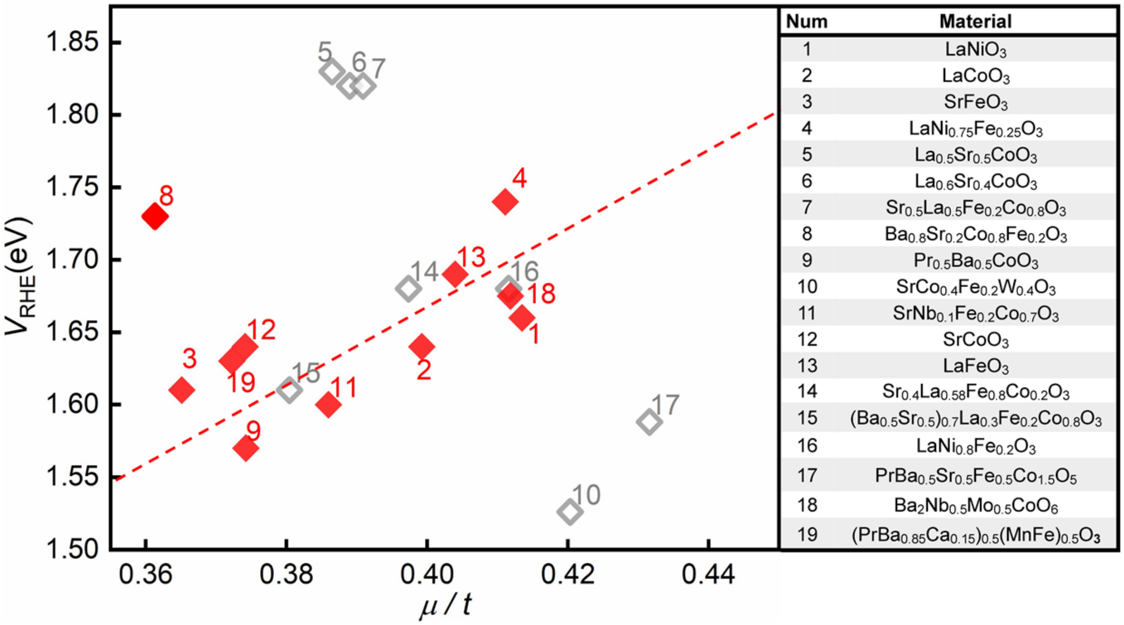

Num	Material
1	$LaNiO_3$
2	$LaCoO_3$
3	$SrFeO_3$
4	$LaNi_{0.75}Fe_{0.25}O_3$
5	$La_{0.5}Sr_{0.5}CoO_3$
6	$La_{0.6}Sr_{0.4}CoO_3$
7	$Sr_{0.5}La_{0.5}Fe_{0.2}Co_{0.8}O_3$
8	$Ba_{0.8}Sr_{0.2}Co_{0.8}Fe_{0.2}O_3$
9	$Pr_{0.5}Ba_{0.5}CoO_3$
10	$SrCo_{0.4}Fe_{0.2}W_{0.4}O_3$
11	$SrNb_{0.1}Fe_{0.2}Co_{0.7}O_3$
12	$SrCoO_3$
13	$LaFeO_3$
14	$Sr_{0.4}La_{0.58}Fe_{0.8}Co_{0.2}O_3$
15	$(Ba_{0.5}Sr_{0.5})_{0.7}La_{0.3}Fe_{0.2}Co_{0.8}O_3$
16	$LaNi_{0.8}Fe_{0.2}O_3$
17	$PrBa_{0.5}Sr_{0.5}Fe_{0.5}Co_{1.5}O_5$
18	$Ba_2Nb_{0.5}Mo_{0.5}CoO_6$
19	$(PrBa_{0.85}Ca_{0.15})_{0.5}(MnFe)_{0.5}O_3$

Supplementary Figure 5 V_{RHE} values, shown according to *μ/t*, of nineteen oxide perovskites listed in Table 6 of Ref. 11 [Energy & Environ. Sci. 12, 442-462 (2019)]. The blank filled points (No. 5, 6, 7, 14, 15, 16) mean that there are no available data, such as loading amount and surface areas in original reference to obtain comparable mass and specific activities. No. 10 had electrolyte concentration of 1.0 M KOH, while others with 0.1 M KOH. No. 17 may have high amount of oxygen vacancy according to its chemical formula. Note that those V_{RHE} values of nineteen oxide perovskites are from eighteen references that are spanning from the year 1979 to 2018. They have different loading amount, surface areas and the values are obtained from different measurements (intrinsic activity or Tafel slopes). It is surprising that a roughly linear correlation to *μ/t* is clearly observed for probably comparable data (red dots). For more details of data, please refer to Ref. 11.

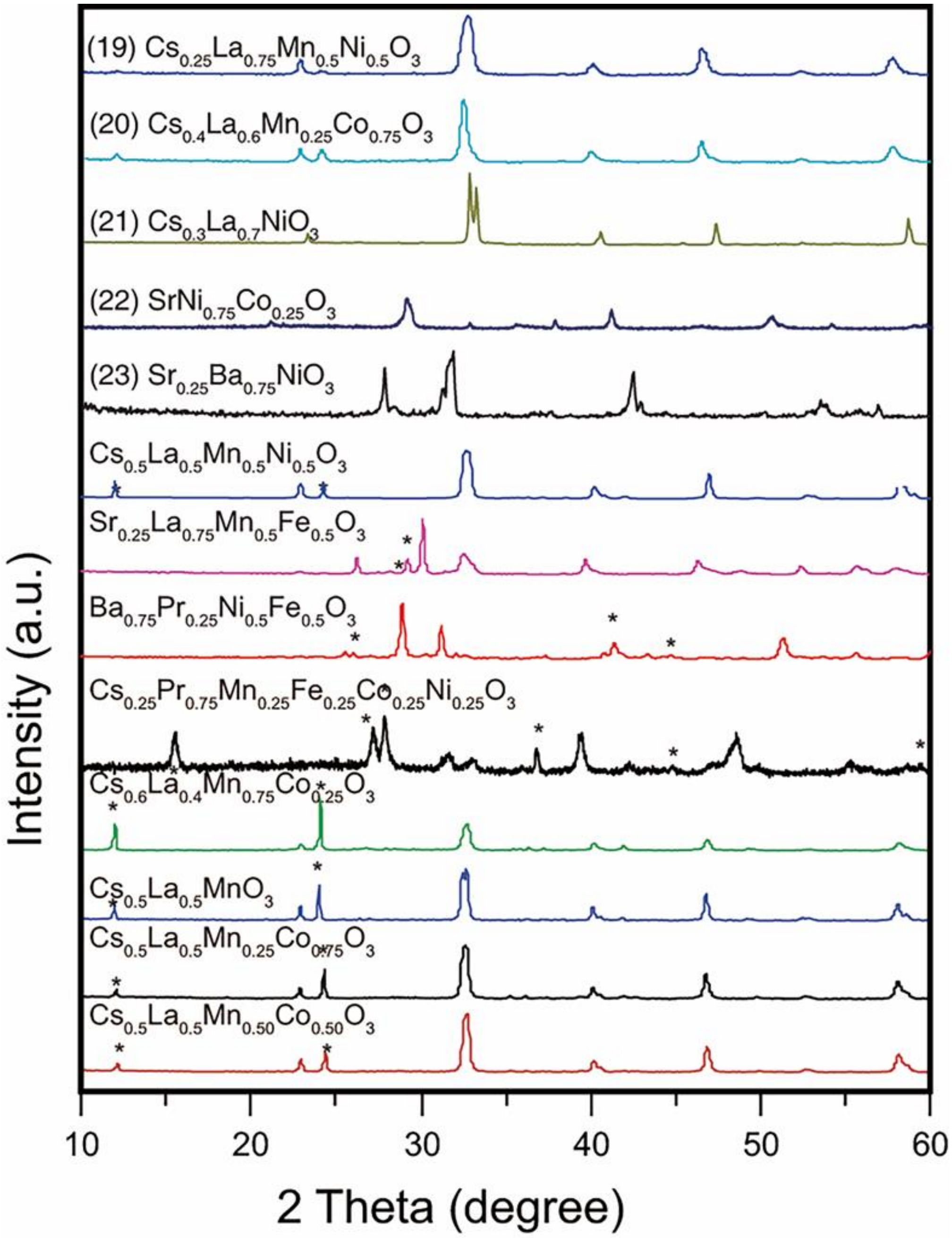

Supplementary Figure 6 PXRD patterns of synthesized oxide perovskite samples containing impurities phases. The impurities are labelled by asterisks. Although all samples show mainly perovskite structures, there remained a significant amount of impurities that were difficult to remove. For example, the peaks at 12° and 24° correspond to the (040) and (111) facets of MnO_x, respectively, and the peak intensities increased with increasing content of Mn and Cs. We speculated that an increase in Cs content may destabilize the structure. The large Mn cations may not easily produce a stable perovskite structure with Cs and La, while the same should be true for Pr with Ba and Cs, due to the large size difference between Pr and Ba/Cs.

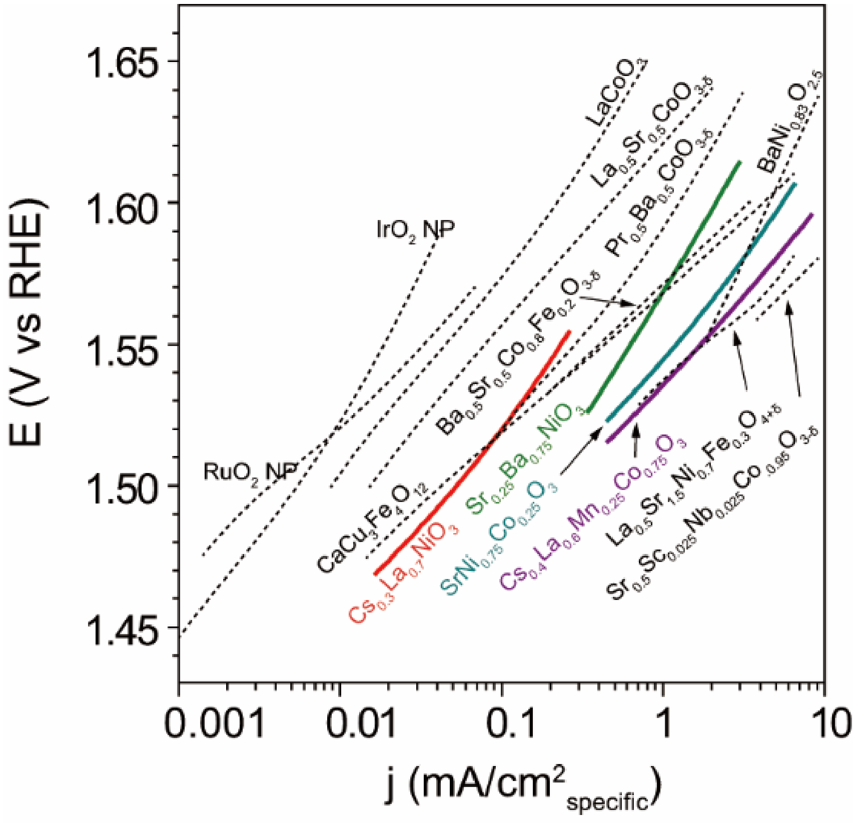

Supplementary Figure 7 The comparison of obtained new perovskite oxides with the state-of-the-art perovskite oxides reported in Ref [8].

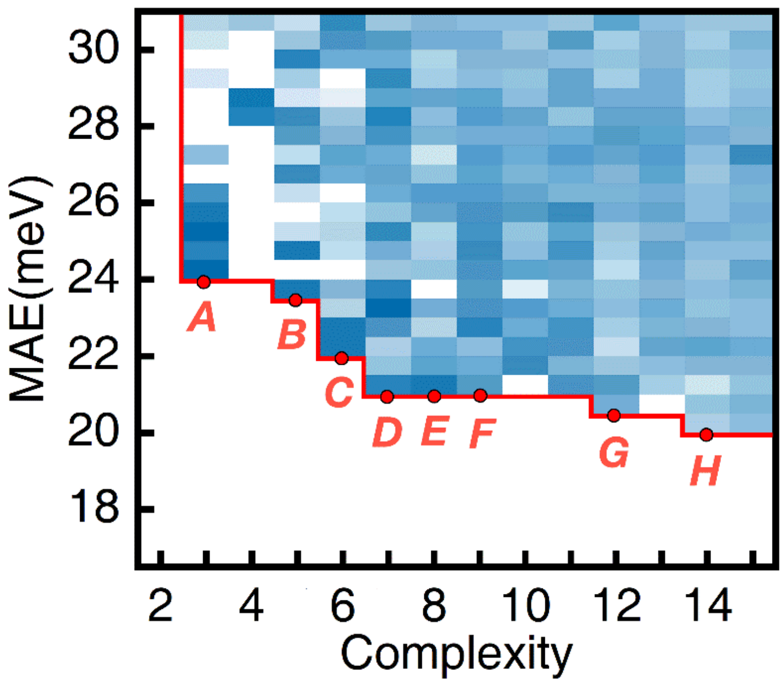

Supplementary Figure 8 Pareto front of MAE vs. complexity of 8,640 mathematical forms in SR process including twenty-three (eighteen conventional and five new) perovskites, shown via density plot. The mathematical forms of A-H were shown in Supplementary Table 3.

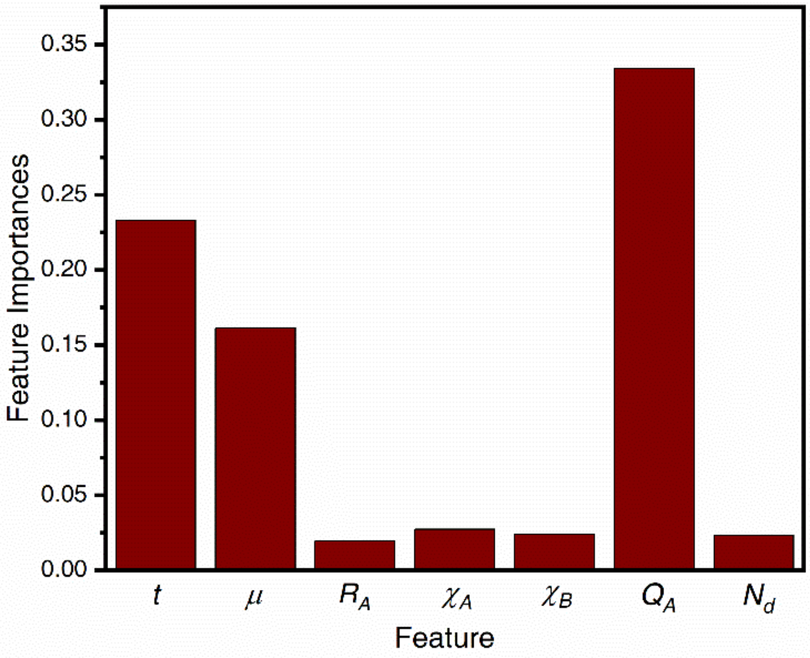

Supplementary Figure 9 Feature importance analysis.

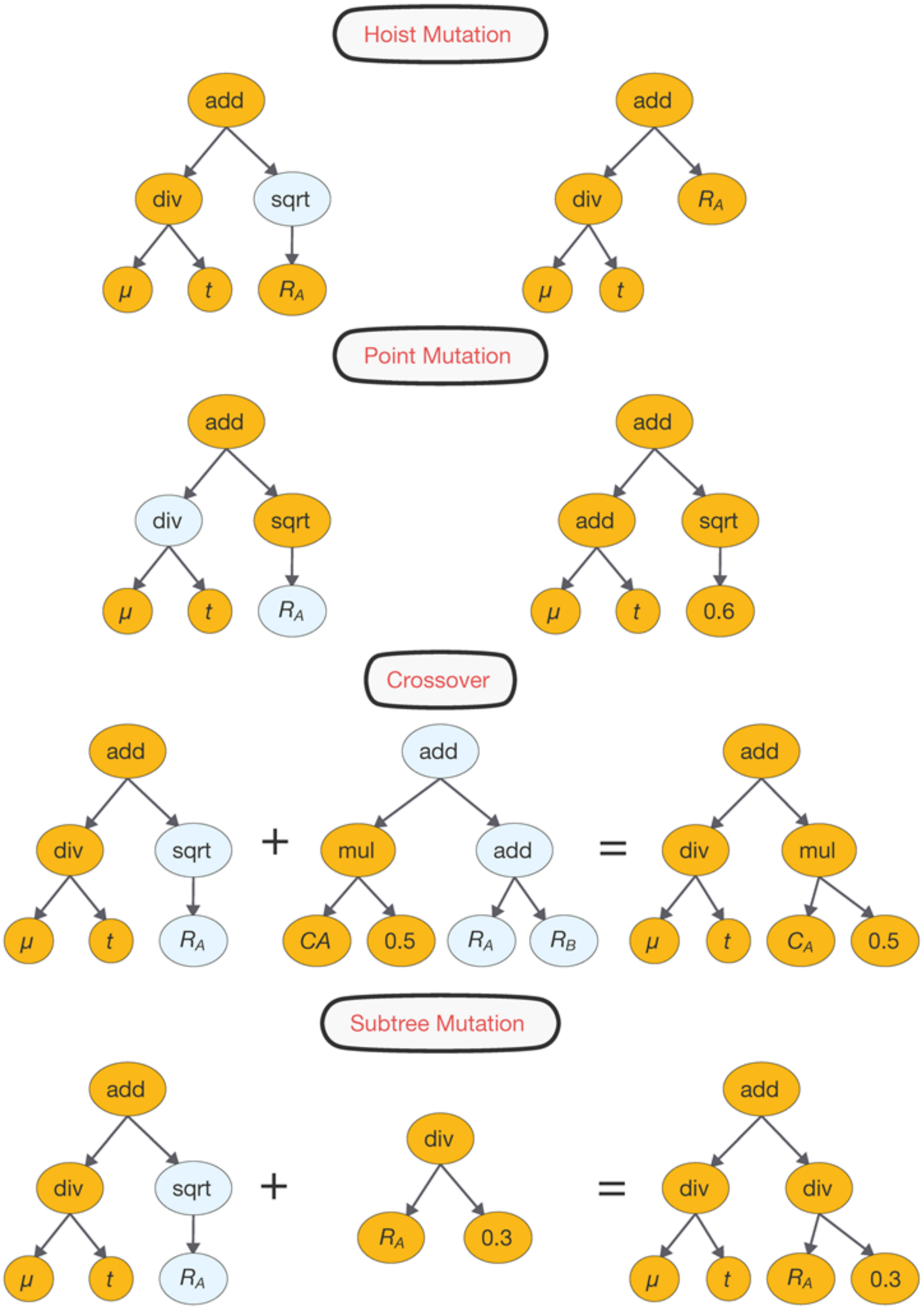

Supplementary Figure 10 The schematic diagrams of four genetic operations used in current genetic programming. a, The hoist mutation method selects a subtree of a randomly selected subtree from the winner of a tournament and replace the previously selected subtree with it. **b,** The point mutation method randomly selects some nodes from the winner of a tournament and replace it with other building blocks. **c,** The crossover method randomly selects a subtree from the winner of a tournament and replace it with a subtree selected at random from the winner of another tournament. **d,** The subtree mutation method randomly selects a subtree from the winner of a tournament and replaces it with a subtree generated at random.

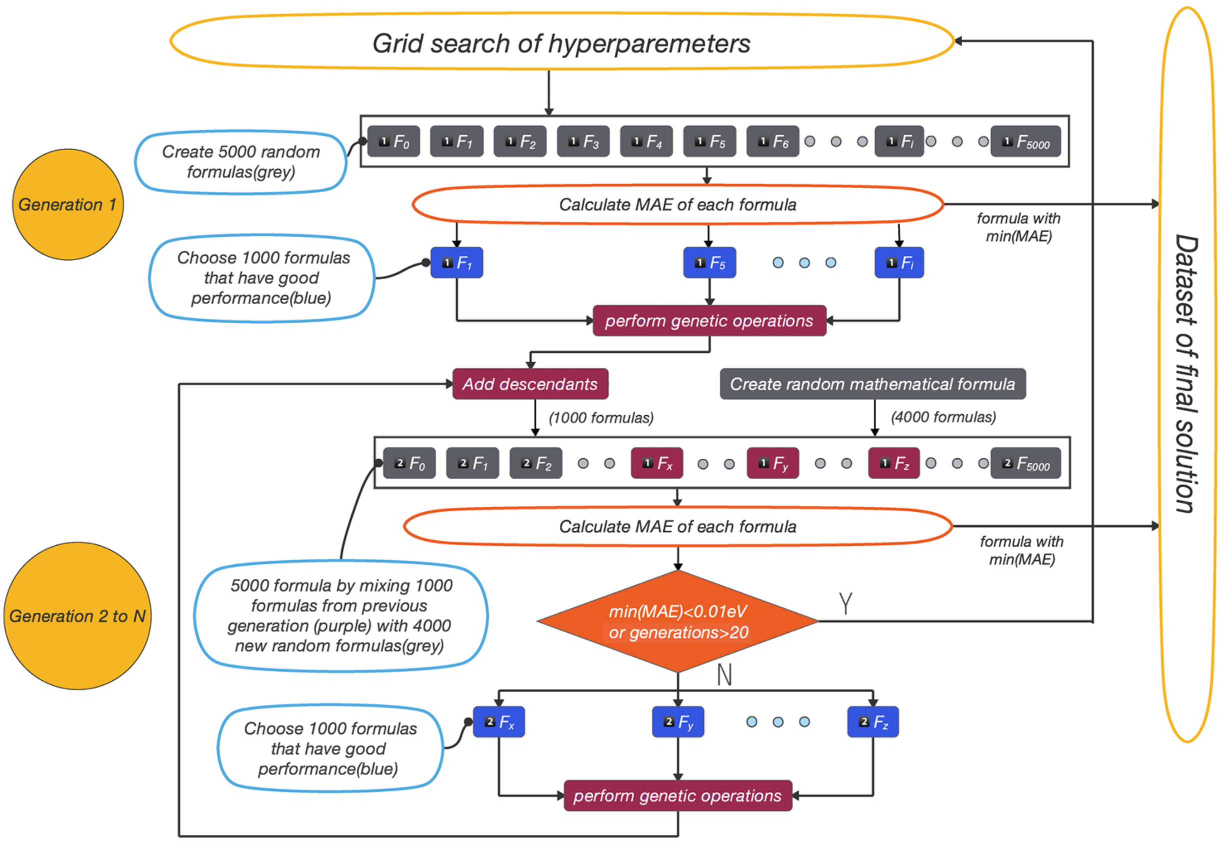

Supplementary Figure 11 Flowchart of symbolic regression based on genetic algorithm. Each generation includes 4000 randomly generated mathematical formulas and 1000 formulas inherited from the previous generation. Each generation provides its best formulas (least MAE) to the final solution sets

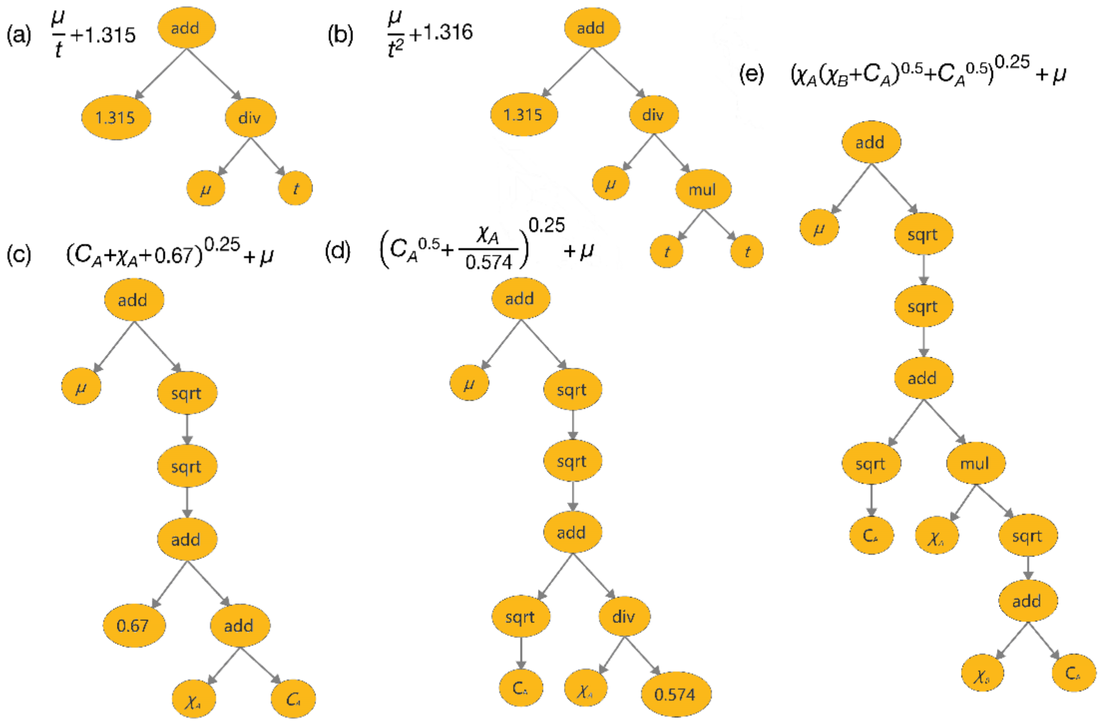

Supplementary Figure 12 The examples of the tree structure for sample mathematical formulas. a, $\mu/t + 1.315$. **b,** $\mu/t^2 + 1.316$. **c,** $(C_A+\chi_A+0.67)^{0.25}+\mu$. **d,** $\left(C_A^{0.5}+\frac{x_A}{0.574}\right)^{0.25}+\mu$. **e,** $\left((\chi_A(\chi_B+C_A)^{0.5}+C_A^{0.5}\right)^{0.25}+\mu$.

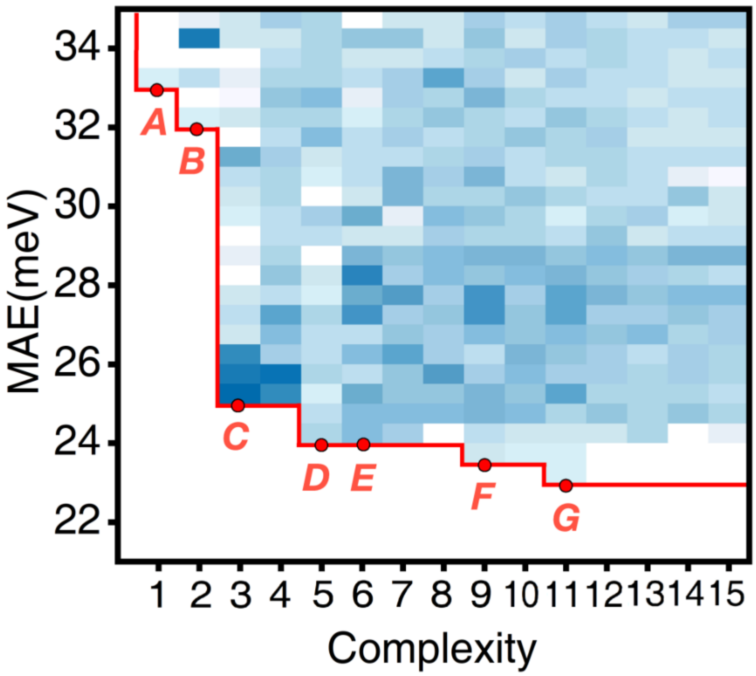

Supplementary Figure 13 Pareto front of SR based on parameters r_A, r_B, N_d, χ_A, χ_B, Q_A

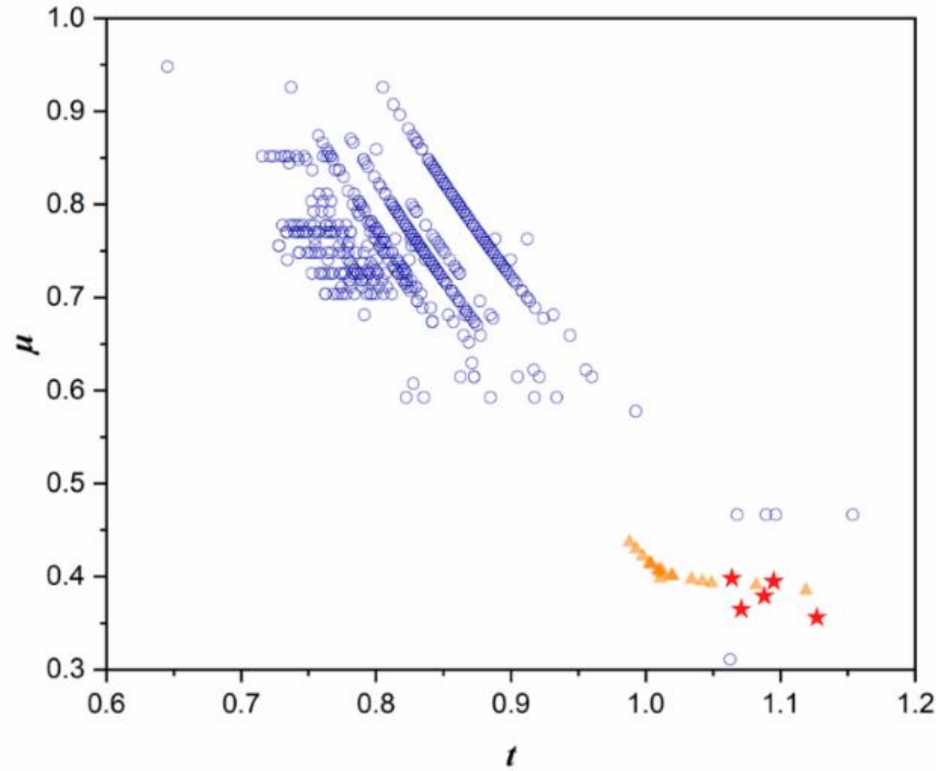

Supplementary Figure 14 The *t*-*μ* map of 534 perovskites (blue circle) with ABO_3 and $A_2B'B''O_6$ formula found in ICSD. The eighteen training samples are marked as orange triangle and five new perovskites in this work are marked as red star.

Supplementary Tables

Supplementary Table 1 The V_{RHE}s of eighteen known oxide perovskite OER catalysts reported in literature at ~ 5 mA cm^{-2} in 0.1 M KOH/NaOH. For comparison, the available V_{RHE}s reported by other groups in literature are also provided.

Materials	V_{RHE} (eV) (This work)	V_{RHE} (eV) (Reference)	V_{RHE} difference (eV)	References
$La_{0.5}Pr_{0.5}FeO_3$	1.725	N/A	N/A	*J. Alloys Comp.*, 2015, **649**, 1260-1266
$PrFeO_3$	1.758	N/A	N/A	*J. Chem. Sci.*, **126**, 517–525
$LaFeO_3$	1.758	1.78	-0.028	*Nano Energy*, 2018, **47**, 199-209
$LaMnO_3$	1.788	1.80	-0.002	*ChemSusChem* 2016, **9**, 1-10
$LaMn_{0.5}Ni_{0.5}O_3$	1.722	N/A	N/A	*Phys. Rev. B*, **65**, 184416
$LaNi_{0.8}Fe_{0.2}O_3$	1.759	1.74	0.019	*J. Mater. Chem. A*, 2015, **3**, 9421-9426
$LaNi_{0.9}Fe_{0.1}O_3$	1.790	1.77	0.02	*J. Mater. Chem. A*, 2015, **3**, 9421-9426
$Sr_{0.25}La_{0.75}Fe_{0.5}Co_{0.5}O_3$	1.718	1.76	-0.042	*ChemSusChem*, 2015, **8**, 1058-1065
$LaNiO_3$	1.707	1.66	0.047	*J. Phys. Chem. Lett.*, 2013, **4**, 1254-1259
$LaMn_{0.5}Cu_{0.5}O_3$	1.774	N/A	N/A	*AIP Conf Proc*, 2014, **1591**, 1630
$LaCoO_3$	1.721	1.64	0.081	*Chem. Mater.*, 2014, **26**, 3368-3376
$La_{0.5}Ca_{0.5}CoO_3$	1.682	1.71	-0.028	*Mater Res Bull* 2000, **35**. 1955–1966
$La_{0.8}Sr_{0.2}CoO_3$	1.688	~1.63	0.058	*Mater. Chem. Phys.*, 1986, **14**, 397-426
$La_{0.4}Sr_{0.6}CoO_3$	1.695	~1.63	0.065	*Mater. Chem. Phys.*, 1986, **14**, 397-426
$BaFeO_3$	1.686	N/A	N/A	*Electrochim Acta*, 2018, **289**, 428-436
$La_{0.2}Sr_{0.8}CoO_3$	1.681	1.70	-0.019	*Int. J. Electrochem. Sci* 2016, **11**, 8633-8645
$SrCoO_3$	1.670	1.65	0.02	*Nature Chem* 2017 **9,** 457–465
$Ba_{0.5}Sr_{0.5}Co_{0.8}Fe_{0.2}O_3$	1.639	1.61	0.029	*Science* 2011, **334**, 1383-1385

Supplementary Table 2 BET surface areas of measured perovskite oxides.

Order	Materials	BET (m^2 g^{-1})
1	$LaMnO_3$	0.311
2	$LaMn_{0.5}Ni_{0.5}O_3$	0.395
3	$LaNiO_3$	0.521
4	$LaMn_{0.5}Cu_{0.5}O_3$	0.372
5	$LaNi_{0.9}Fe_{0.1}O_3$	0.468
6	$LaNi_{0.8}Fe_{0.2}O_3$	0.567
7	$LaFeO_3$	0.258
8	$La_{0.5}Pr_{0.5}FeO_3$	0.383
9	$PrFeO_3$	0.447
10	$LaCoO_3$	0.492
11	$La_{0.5}Ca_{0.5}CoO_3$	0.762
12	$La_{0.8}Sr_{0.2}CoO_3$	0.749
13	$Sr_{0.25}La_{0.75}Fe_{0.5}Co_{0.5}O_3$	0.263
14	$La_{0.4}Sr_{0.6}CoO_3$	0.482
15	$La_{0.2}Sr_{0.8}CoO_3$	0.273
16	$SrCoO_3$	0.483
17	$Ba_{0.5}Sr_{0.5}Co_{0.8}Fe_{0.2}O_3$	0.300
18	BaFeO3	0.704
New Materials		
19	$Cs_{0.25}La_{0.75}Mn_{0.5}Ni_{0.5}O_3$	0.527
20	$Cs_{0.4}La_{0.6}Mn_{0.25}Co_{0.75}O_3$	0.308
21	$Cs_{0.3}La_{0.7}NiO_3$	0.317
22	$SrNi_{0.75}Co_{0.25}O_3$	0.314
23	$Sr_{0.25}Ba_{0.75}NiO_3$	0.237

Supplementary Table 3 The eight mathematical formulas at the Pareto front in Supplementary Figure 8.

Point	Formulas	MAE (eV)	Number of parameters	Complexity
A	$\frac{1.751}{t}$	0.0245	1	3
B	$\frac{1.672}{t}+0.681$	0.0237	1	5
C	$\frac{1.093}{t}+\mu^{0.5}$	0.0224	2	6
D	$1.804\frac{\mu}{t}+1$	0.0213	2	7
E	$1.073\frac{\mu}{t^2}+1.295$	0.0211	2	8
F	$\frac{\mu}{t(R_A-\mu)}+1.315$	0.0214	3	9
G	$\frac{\mu}{t}+\left(\left((Q_A-R_A)^{0.5}\right)^{0.5}+0.744\right)^{0.5}$	0.0205	4	12
H	$\frac{\mu}{t}+\left((\frac{1.899}{t}+1.005Q_A{}^{0.5})^{0.5}\right)^{0.5}$	0.0203	3	14

Supplementary Table 4 ICP-AAS analysis of $Cs_{0.4}La_{0.6}Mn_{0.25}Co_{0.75}O_3$ and $Ba_{0.75}$ $Sr_{0.25}NiO_3$ samples before and after OER test. ICP-AAS indicates the slight leaching of “A”-site atoms, which may explain the current variations during the stability test [Adv. Mater. 31, 190083 (2019)].

**Samples**	**$Cs_{0.4}La_{0.6}Mn_{0.25}Co_{0.75}O_3$**				**$Sr_{0.25}Ba_{0.75}NiO_3$**		
	Metal ions concentrations (ppm)						
**Elements**	Cs	La	Mn	Co	Sr	Ba	Ni
**Before cycling**	19.91	30.07	12.48	37.54	11.7	38.03	50.27
**After cycling**	18.25	30.62	13.03	38.11	11.29	37.77	50.95

Supplementary Table 5 The formulas on the Pareto front of Supplementary Figure 13.

Point	Formulas	MAE (eV)	Complexity
A	$0.162+r_B$	0.0333	1
B	$(1.455\chi_A+1.359)^{0.5}$	0.0322	2
C	$(Q_A^{0.5}+1.358)^{0.5}$	0.0256	3
D	$((Q_A+r_B)^{0.5}+1.157)^{0.5}$	0.0248	5
E	$(Q_A^{0.5}+(2.225r_A)^{0.25})^{0.5}$	0.0246	6
F	$\left(\frac{(1.733r_A)^{0.5}}{\chi_B^{0.25}}+Q_A^{0.5}\right)^{0.5}$	0.0235	9
G	$\left(\frac{((r_B+1.123)r_A)^{0.5}}{\chi_B^{0.25}}+Q_A^{0.5}\right)^{0.5}$	0.0232	11

Supplementary References

1. Wang, Y., Wagner, N. & Rondinelli, J. M. Symbolic regression in materials science. MRS Commun. 9, 793–805 (2019).
2. Schmidt, M. & Lipson, H, Distilling Free-Form Natural Laws from Experimental Data. *Science* **324**, 81-85 (2009).
3. Koza, J. R. *Genetic Programming: On the Programming of Computers by Means of Natural Selection.* (*MIT Press, Cambridge, MA*). (1992).
4. Forrest, S, Genetic Algorithms - Principles Of Natural-Selection Applied To Computation. *Science* **261**, 872-878 (1993).
5. Butler, K. T., Davies, D. W., Cartwright, H., Isayev, O. & Walsh, A, Machine learning for molecular and materials science. *Nature* **559**, 547-555 (2018).
6. Stephens, T. gplearn. https://gplearn.readthedocs.io/en/latest/intro.html.
7. Wei, C. *et al*. Recommended Practices and Benchmark Activity for Hydrogen and Oxygen Electrocatalysis in Water Splitting and Fuel Cells. *Advanced Materials* **31**, 1806296 (2019).
