# Supplementary material for: Simple descriptor derived from symbolic regression accelerating the discovery of new perovskite catalysts
Source: Nat Commun. 2020 Jul 14;11:3513. doi: 10.1038/s41467-020-17263-9 (PMC7360597; doi:10.1038/s41467-020-17263-9)
Supplement: Supplementary file 3 — Description of Additional Supplementary Files [file 41467_2020_17263_MOESM3_ESM.pdf]

Description of Additional Supplementary Files

File name: Supplementary Data 1
Description: Exact values of data points (at current density 50 μA/cm^2) in Fig. 2a.

File name: Supplementary Data 2
Description: Exact values of data points (at current density 5 mA/cm^2) in Fig. 2a.

File name: Supplementary Data 3
Description: Exact values of data points (at current density 10 mA/cm^2) in Fig. 2a.

File name: Supplementary Data 4
Description: Exact values of data points (at current density 15 mA/cm^2) in Fig. 2a.

File name: Supplementary Data 5
Description: Exact values of data points (at current density 20 mA/cm^2) in Fig. 2a.

File name: Supplementary Data 6
Description: Screened oxide perovskites. The new oxide perovskites are listed in the sequence of μ/t. In the table, as a typical example,
'Cs+0.5La3+0.5Mn3+0.25Mn4+0Fe3+0.75Fe4+0Co3+0Co4+0Ni3+0Ni4+1.0'
means '$Cs^{+}_{0.5}La^{3+}_{0.5}Mn^{3+}_{0.25}Mn^{4+}_{0}Fe^{3+}_{0.75}Fe^{4+}_{0}Co^{3+}_{0}Co^{4+}_{0}Ni^{3+}_{0}Ni^{4+}_{1.0}O_3$'

File name: Supplementary Data 7
Description: The ionic and compositional data used for screening of new oxide perovskites. The table shows examples of the construction of twenty-three perovskite oxides.
